# Introductory Lecture to a Course on Obstetrics, Delivered in Jefferson Medical College, November 4, 1841

**Published:** 1841-12-04

**Authors:** 


### BIBLIOGRAPHICAL NOTICE.

*Introductory Lecture to a Course on Obstetrics, delivered in Jefferson Medical College, November* 4, 1841. By Charles D. Meigs, M.D., Professor of Obstetrics.

The number of introductory lectures on our table is this year unusually large. This accumulation may be, in part, attributed to the impetus given to the medical school of Philadelphia by the almost entire reorganization of the Jefferson Medical College. The inaugural addresses of the newly appointed professors are of course expected for publication, while their seniors deem the occasion, as we have seen, a fair one "to speak out and be heard for themselves." We have noticed this series of introductory lectures at considerable length—many of them as the maiden efforts of distinguished gentlemen, and all of them as having special interest from the change in the relative position of the schools which they represent.

The lecture of Professor Meigs is altogether worthy of the high reputation of the author. It is an elegant composition—eloquent, and appropriate to the occasion. Appearing for the first time in the station of a public lecturer, Dr. Meigs has naturally introduced his remarks to his class with some allusion to himself; and, with a brief and modest notice of his own early career, he has gracefully blended highly finished and beautiful sketches of the great men of his pupilage. The doctor commenced the study of medicine in 1809, with the late Dr. Fendall of Augusta, in Georgia, whose name he mentions in terms of warm respect. He completed his education in the University of Pennsylvania, and of the professors of that day he draws hese glowing portraits. First, Rush—the American Sydenham:

"The eloquent accents of that venerable man seem to fall upon my ears even now, when I turn back my thoughts to those young days filled with aspiring hopes and fond anticipations of success and professional distinction. I see him now, surrounded by 500 young men, my fellow students and fellow citizens from every part of our wide-spread country, each one gazing intently upon that reverend countenance, wrinkled with age it is true, but still ruddy with temperance, and radiant with the smile which showed how charming the divine philosophy that sat enthroned upon a brow of the rarest benignity and beauty. I see that good old man erect himself in his chair, upon which, on account of his great age, he was accustomed to sit at his lecture. He puts back his glasses on his forehead—he rises from his seat, and leans with his aged hands upon the desk—he looks abroad over the whole mass of faces and says, "gentlemen, *silence!* I rise from my seat for a special object—I desire you all to remember, that upon this day, I stood up before you while endeavouring to impress upon your minds the necessity of opposing the very beginnings of disease—in order that I might pronounce these two words in your ears—OBSTA PRINCIPIIS, OBSTA PRINCIPIIS." Those words sunk ineffaceably into every man's memory—you hear me repeat them at the distance of twenty-nine years; and it was by such methods as this—by some graceful wave of the hand, by some forcible gesture of the body, by some most apposite illustration, that he endeavoured always to impress deep into the plastic material before him, that signet of his intellectual power whose traces are still visible in the Mens Medica of these United States—which I firmly believe, is extending its nature and kind, as a good leaven that leaveneth the whole lump, beyond the Atlantic wave."

Barton:

"There, too, I heard the lessons of the fiery Barton. He had a head that seemed chiselled as by a sculptor, so firm and unwavering was it in its resolute expression. He came there scrupulously dressed, and exactly punctual, to pour the rich and fertilizing stream of his discourse, while his face often became the express image of his sentiment, as he felt the warm and generous glow of Linnæus' zeal at the prospect or the hope of some new medicinal herb. When he told us of opium,—of its talismanic properties, and its baleful powers, the tears coursed down his sympathetic cheeks and ours, as he related the history of the immortal Brown, his early friend—his meteoric fame—his shining intelligence—his dark, and dreary, and dismal fall and death. And then he would gather himself up again to criticise the doctrines of Cullen, and Murray, and many others, and to urge, urge, urge upon us the results of his own experience in the therapeutic properties of the preparations of lead, or the nature of American medicines; whilst it was a delight and an honour to sit at his feet and listen, as he poured his lay, almost poetical, over the dry and barren fields of the Materia Medica."

But there was a Gamaliel at whose feet one might be deemed happy to sit even all the day long:

"Look at that great amphitheatre, crowded to the outermost ring. No stamping of noisy feet indicated the impatience of a crowd for the arrival of a tardy master. No—at the *point* of time he entered the area. He came, with that cold eye, which you could neither bear nor forbear—its light was different from that of common men. He came with that face of pentelic marble—that hair powdered and dressed in the most finished manner—that blue coat with its metal buttons, closed on his breast on account of his delicate health. There he stood silent for a moment. You would as soon think to cheer a statue, or applaud at the marble features of a corpse, as to have raised your voices in praise or blame where Dr. Physick stood. He opened his mouth after a cold salute, and from thence proceeded choice words of wisdom, which we were too anxious to gather up in our garners of note books, to stop for a moment to see what other men were doing, or imagine what they were thinking, for so great was our trust in what he should say, that we received it as a gospel; and truth to speak, no word of folly or frolic did I ever hear proceeding from the lips of that great man, who deemed the business of dealing with men's lives and teaching others to do so, one of such solemn and dreadful import that there was no place in it for glee or laughter; and so he acted, and so he always looked—he lived so, and he died in that belief. Dr. Physick was a very great man, gentlemen: You had an indefeasible tendency to stand uncovered in his presence. There was a spotless purity in his character, so that he walked as in a bright cloud of moral truth and beauty. Apollo, the god of physicians, seemed to have inspired the nobleness of his countenance, and to have imparted somewhat of the *mens divina* to his whole moral constitution. You and I may live long, gentlemen, yea, and our children after us, before so rare a combination of great and admirable qualities shall again conspire to produce the perfect pattern and model of a surgeon and physician."

Wistar, the beloved of the class:

"But why have I not yet spoken of *him*, the beloved of the class! By what epithets shall I attempt to particularize those singular good qualities, which, by a happy conspiration, united to make up the character of that good old man—the idol, the darling of the classes? Do you not see that powdered head of his, with its well-adjusted locks and queue? Dr. Wistar enters the area, followed by a cloud of witnesses, bowing often, and rubbing his hands, and with a face on which sat a pleased and yet bashful expression—a mixture of emotions which gave it a most peculiar character—chiefly delightful, however. He came there amidst sounds of greeting, and the wreathed smiles and looks of exchanged congratulations of the superimposed circles. Men witnessed his entrance as they witness the completeness of preparation for some great feast; there was a satisfied feeling already, like that with which a company inhale the rich perfumes and odours of a feast that is set.

He lifted his hand, and in a very short, quick expression, he said, *Gentlemen!* Henceforth all was still—a profound silence, broken only by the arrival of some tardy student, which was regarded as a wrong done to the whole company, and a rudeness to be visited by frowns, or even more decided marks of disapprobation, particularly if the white-haired teacher should stop in his career to look around the sky-parlours. Ah, gentlemen, those were the halcyon days of medical instruction!—days ever to be remembered. But those good men are gone off the stage of the world. The eloquent voice of Rush is silent where he lies yonder in his grave; and Wistar sleeps among the undistinguished dead of his sect, in that ground to which I followed his remains—one of a vast concourse of his fellow citizens, treading with mournful steps, and slowly, the way taken by the dead body of a public benefactor. I felt that day—grieved as I was to part for ever with one who had gained my whole esteem and reverence—that I was honoured in being a physician, for my profession was exalted and honoured in his life, and by the public testimonials to his worth and many virtues rendered at his death."

Dorsey:

"The beautiful Dorsey, with a face as bright as the morning, and open as noon day. An ambition of the highest reach urged him onward in a career that was nobly run, and would have carried off the highest prize, had he been spared to the country. Conquering, by most arduous struggles, certain natural impediments of his elocution, he had just attained the perfect victory. He had just stepped on a lofty stage of action, when the angel of death struck him down too, that he might, though young, belong to that great age of American Medicine. He came not down to our times, but was gathered to his brethren and his like. He sleeps here among them. The American Sir Astley, is a title which he deserved, not more by the graces of a most ornate mind and manners, than by the great surgical skill and renown which he so early vindicated to himself."

And James:

"Dr. Thomas Chalkley James, Professor of Midwifery in that day—a member of the Society of Friends,—a *good man*. There are many persons here, I suppose, who remember the quiet, calm, gentle, modest style with which he came out into the rotunda to meet us in the afternoons. He brought there written lectures filled with learning, ransacked from the whole stores of that time, and arrayed for us into an order and a show that made them always delightful,—garnished, as they were, with apposite clasical citations, whether from the ancient or modern authors. He brought, too, the results of a great experience in practice. He brought there, also, his modesty, which never left him from his earliest youth, and which frequently sent the mantling blood over cheeks and brow to testify that he had the deepest sense of the delicacy of the task assigned to him—that of exposing to hundreds of young men, those trembling secrets of the lying-in chamber, which he had blushed to learn, and which he more redly blushed to tell. Take him all-in-all, and you shall search long and far before you shall find a more honourable, upright gentleman—a riper scholar—a better teacher, or a better man."

Largely as we have quoted from this lecture, we cannot resist the temptation to offer another extract. It is not a portrait, but a scene. From himself and his teacher, the Doctor has passed to his subject proper, and after dwelling upon the importance and responsibilities of the obstetric art, he thus vividly draws the picture of a lying-in-room.

"Did you ever," he addresses the student, "set your feet inside of such a sanctuary as that! Tread softly! Here is the door. Look, how beautifully clean those walls and ceiling are; a lady's glove is not fairer. See that nicely carpeted floor! those window curtains of beautiful fashion, to exclude the flaunting lights of the world, which must have no intrusion here, where the lights should be soft and dim. Observe that bed made up, with its snowy pillows, and its beautiful hangings of silk damask! Take a white kid glove upon your hand, and see whether you can find the smallest particle of dust, or the least remainder from the last sweepings and dustings of the well-bred housemaid. There stands a wardrobe of the richest form and materials, burnished and bright; and here—come on this side!—in the second drawer from the top of this exquisite bureau, which I open for you, she has secretly, but with sweet smiles, the harbingers of a new hope and a new happiness for this whole house, put carefully away all this beautiful array of worked caps and frocks and bands and slippers, with colours, as you see, like a bright parterre, of ribands of every hue—blue, and pink, and white, and green, and red, and purple,—all adapted by her own taper fingers when alone, or the welcome gifts of her many loving and anxious friends. Here they are! and no man's eye has seen them before, waiting to be selected by the nurse, or by some good aunt, when that happy hour shall have come that brings with it deliverance and safety, congratulations, sobs of happiness too great for the bosom to contain, and the sweetest sensations of human complacency in the finished, concluded, and blissful result of a long-tried and holy love.

Such, and so careful and recherché, are the preparations and arrangements for an expected confinement, that it is really quite an agreeable spectacle to look upon in reference to the moral associations connected with these events and which lend to them a charm so truly poetical, that the ancients were happy to reduce them into a physical and sensible and visible expression, by the invention of the divine Pysche, the soul, the object of such tender affection by her lover. A true and sacred affection, purified and mundified of the dross and corruption of the human nature, must be like that of this exquisite fable—a purely pyscological ens or quality.

But let me proceed with my story. Let us look farther into some of the events of the profession you are about to make!

The first signal is given; the hour anxiously looked for, and even longed for with fear, has come at last, and the signal of its arrival is pain. She is gone to that chamber, and she trembles and grows pale, that young and beautiful thing! Yes! she trembles, and is amazed at the new-born sensations that have seized upon her frame. Her consciousness, her teleological sense, teaches her that a crisis is at hand; and the whole house is aroused to a sort of excitement and perturbation, which soon is communicated to those friends and relatives whose inquiries have been lately repeated more and more frequently. The busy, kind-hearted, and bustling nurse is summoned where she sits at home, with wisdom and prudence brooding on her brow, waiting for the summons, as she has been every day for a week or more. Her little bundle is soon made up, her trunk is packed, and away she hies to carry with her some assurance of safety, much positive comfort, and the hope of a most prosperous getting up, at least.

The pain comes again—and again it grows severe, and every eye is turned with tenderness and compassion upon that young and beloved girl, when she sits in the great arm chair and bears or tries to bear that which cannot be borne. At length the plot thickens; events are at hand which require the controlling and guiding hand and the instructed head of the physician. Let us stop here. That beautiful creature, in the great crisis of nature, is an only daughter,—the object of the concentrated affections of parents and relatives. To form her manners—to lend grace to her natural beauties—to guide her flying fingers over the cords of the harp or piano—to teach her strange tongues—to attune her voice to the composition of Boyeldieu, of Mozart, of Rossini, and Bellini—to adorn her person—thousands of pounds have been expended, and sleepless eyes have often watched and waited for her return from the party or the ball, the scene of the triumphs of her beauty; and here she is at last, caught in the toils, destined to pass through pains like those of a crucifixion, and to incur hazards against which the Church has ordained a set form of prayers. What vast interests—interests of the heart—are here put upon the hazard of the die; and you, sir—or you—or you—are summoned to stand betwixt her and those agonies, those dangers, or that death, which stands with his cold and bony hand poising the dart aimed against that priceless life! Will you take all this responsibility upon yourself? Will you go there to that sacred apartment, where man never trod before, save the privileged one? and are you ready to say, come what may, here am I! There is nothing here with which I am not familiar as with household phrases and the most trite occasions. Beware what you do! Enter not into that sacred precinct, unless with the loins of your mind girded—with your lamp of knowledge trimmed and burning—with that gem of the mine flashing on your brow—knowing, feeling, conscious that you are equal to the grave duty you assume. If you can truly say, I know that duty—I know it as well as it can be known by mortal man—then go and stand like the priest of Lucina, and your offering of science, of skill, of sagacity, of humanity, will be an accepted one; the victim, bound and crowned for the sacrifice, shall go free like another daughter of Clytemnestra at Aulis, for your vow is fulfilled.

Yet again let us take a further view of these mysteries. You arrive, and a feeling of renewed security comes along with you. You speak words of calm and assured confidence. You exhort that lady to bear the pain with patience and hope, and you take your seat to observe the progress of this curious scene.

The labour goes on, and having acquired the needful information, you make your announcement of a favourable and speedy termination of the distress of that beautiful creature. But you are disappointed; the affair lingers—she becomes restless—she tosses from side to side; groans and cries proceed with sobs from that bosom which never felt a pang before; and that countenance is flushed and swollen, which, before, the winds of heaven had never visited roughly. What is the matter! "The pain—the pain—the pain! I shall never recover—I cannot endure all this—my head aches!" There you sit, sir—for you know that the face is to be flushed in labour, and the pain is great and the patient naturally restless. She rises on her elbow and says: "Doctor, what is the matter? I cannot see you. Oh, how my head turns!—how it aches!" "Never mind it; have patience. You are nervous—don't be nervous—the child is almost born. You will soon be well." There, sir, you have fallen into an impassible and bottomless gulf of regrets—you have plunged that lovely woman headlong down, and she is now irretrievably destined to lie, as Shakspeare says, in cold obstruction and to rot—and that happy home is desolate—that temple of peace is broken down, for its idol has fallen, and shall disappear for ever from the gaze of those eyes that have been fondly bent upon it for so many years.

The child is not born; but in an instant—in the twinkling of an eye—horror has seized upon all the occupants of that chamber. The sounds of running feet—the accents of wo, wo, wo, are heard, and a wild confusion has suddenly succeeded to the quiet of that chamber, which before was broken only by the moans of that lady, approaching to the supposed consummation of her happiness. A loud and hissing sound, as the hiss of a thousand snakes, issues from her now livid and distorted mouth—those eyes, which it were heaven to look upon before, are rolled in opposite directions or protruded to bursting from their wide-open sockets. That brilliant brow is overspread with a dark livor; and that breath, like new mown hay, is but a succession of frightful explosions, scattering foam and blood in every direction, dabbling the bright hair, or flecking the exposed and agitated bosom; while the wildest and most fearful convulsions wrench and torment those beautiful limbs; and there she lies, that lovely one, as if the prey of fiends, delighting to tear and rend and reduce that image of grace to their own frightful and abominable deformity. What is all this, gentlemen? It is no fancy sketch—it is a sober and imperfect representation of the horrors of a lying-in-room, where a beautiful woman is seized with puerperal convulsions—a common occurrence—and which, I appeal to the respectable gentlemen here, is among the dreadful events of the life of a surgeon accoucheur. You ought to have prevented it, sir, or you ought to seize with a lightning rapidity upon the indications. You ought to be able to say to all this confusion and tumult, "Peace, be still; I am here, I shall extend all the relief that is possible. All the resources of art are in my hands: peace, be still." Here is the demand for that knowledge which men *pray* for. There is no gibe for the physician now. No: he alone has the skill and the knowledge which cannot be begged, borrowed or bought—it must be earned."

The length of these quotations is much beyond our prescribed limits, but we believe our readers will not regret the space allotted to them.

